# Genetic Adaptation vs. Ecophysiological Plasticity of Photosynthetic-Related Traits in Young *Picea glauca* Trees along a Regional Climatic Gradient

**DOI:** 10.3389/fpls.2016.00048

**Published:** 2016-02-03

**Authors:** Lahcen Benomar, Mohammed S. Lamhamedi, André Rainville, Jean Beaulieu, Jean Bousquet, Hank A. Margolis

**Affiliations:** ^1^Faculté de Foresterie, de Géographie et de Géomatique, Centre D'étude de la Forêt, Université LavalQuebec, QC, Canada; ^2^Direction de la Recherche Forestière, Ministère des Forêts, de la Faune et des ParcsQuebec, QC, Canada

**Keywords:** white spruce, climate change, assisted migration, local adaptation, acclimation, functional traits, mesophyll conductance, photosynthetic capacity

## Abstract

Assisted population migration (APM) is the intentional movement of populations within a species range to sites where future environmental conditions are projected to be more conducive to growth. APM has been proposed as a proactive adaptation strategy to maintain forest productivity and to reduce the vulnerability of forest ecosystems to projected climate change. The validity of such a strategy will depend on the adaptation capacity of populations, which can partially be evaluated by the ecophysiological response of different genetic sources along a climatic gradient. This adaptation capacity results from the compromise between (i) the degree of genetic adaptation of seed sources to their environment of origin and (ii) the phenotypic plasticity of functional trait which can make it possible for transferred seed sources to positively respond to new growing conditions. We examined phenotypic variation in morphophysiological traits of six seed sources of white spruce (*Picea glauca* [Moench] Voss) along a regional climatic gradient in Québec, Canada. Seedlings from the seed sources were planted at three forest sites representing a mean annual temperature (MAT) gradient of 2.2°C. During the second growing season, we measured height growth (H2014) and traits related to resources use efficiency and photosynthetic rate (*A*_max_). All functional traits showed an adaptive response to the climatic gradient. Traits such as H2014, *A*_max_, stomatal conductance (*g*_*s*_), the ratio of mesophyll to stomatal conductance, water use efficiency, and photosynthetic nitrogen-use efficiency showed significant variation in both physiological plasticity due to the planting site and seed source variation related to local genetic adaptation. However, the amplitude of seed source variation was much less than that related to plantation sites in the area investigated. The six seed sources showed a similar level of physiological plasticity. H2014, *A*_max_ and *g*_*s*_, but not carboxylation capacity (*V*_*c*max_), were correlated and decreased with a reduction of the average temperature of the growing season at seed origin. The clinal variation in H2014 and *A*_max_ appeared to be driven by CO_2_ conductance. The presence of locally adapted functional traits suggests that the use of APM may have advantages for optimizing seed source productivity in future local climates.

## Introduction

In boreal regions, climate is the main factor controlling the spatial distribution of tree species (Davis and Shaw, 2001). For most widespread tree species, natural selection processes operated over thousands of years, has led to local adaptation and differentiation of populations along climatic gradients across their natural range (e.g., Andalo et al., 2005; Aitken and Whitlock, 2013; Savolainen et al., 2013). This climate-related adaptive differentiation along geographic gradients has been demonstrated through provenance tests for several boreal tree species in eastern Canada (Li et al., 1997; Beaulieu et al., 2004; Andalo et al., 2005; Lu et al., 2014; Rossi and Bousquet, 2014; Yang et al., 2015). Therefore, the actual pace of climate change is expected to induce a spatial mismatch between locally adapted populations and their optimal climatic conditions to which they have historically adapted, leading potentially to local maladaptation (Gray and Hamann, 2011; Aitken and Whitlock, 2013). Such a trend could result in a reduction in forest health and productivity, as well as in ecosystem services and the resources they provide (Andalo et al., 2005; Aitken et al., 2008; O'Neill et al., 2008). Given that the evidence for global climate change is now unequivocal (Aitken and Bemmels, 2015), the paradigm of “local seed is the best” may no longer be appropriate for forest plantation programs under a changing climate (Gray and Hamann, 2013; Rainville et al., 2014; Aitken and Bemmels, 2015).

Assisted population migration (APM) is the intentional movement of populations to areas that are expected to harbor future environmental conditions similar to those at their geographical location of origin (Aitken and Whitlock, 2013; Ste-Marie, 2014). APM has been proposed (with current implementation planning in Canada) as a proactive adaptation strategy to maintain forest productivity and reduce the vulnerability of forest ecosystems to climate change (Gray and Hamann, 2011; Pedlar et al., 2011; Aitken and Whitlock, 2013). Despite the potential benefits of this proactive approach, APM implementation should nevertheless be preferentially preceded by trial tests supplemented by the acquisition of more exhaustive ecophysiological knowledge (Isaac-Renton et al., 2014). This is particularly important at the juvenile phase for appropriate adaptation and establishment of seedlings under different site conditions.

Climate-based seed transfer models based on the relationship between climatic parameters such as mean annual temperature (MAT) and summary quantitative traits such as height growth have been widely used to predict optimal climate-transfer distances for APM (Andalo et al., 2005; Wang et al., 2010; O'Neill and Nigh, 2011). These empirical models were generally based on previous provenance test results and transfer distances varying from about 1.5–4°C (MAT) in the boreal region (Andalo et al., 2005; Wang et al., 2010; Pedlar et al., 2011; Yang et al., 2015). However, these models did not take into account several considerations which may increase the risks associated with seed source transfer (O'Neill et al., 2008; Isaac-Renton et al., 2014). These include (i) the higher temporal variability of climatic conditions in boreal regions resulting from climate change, which may affect populations fitness depending on whether or not an extreme event occurs during provenance testing (particularly at the juvenile growing stage); (ii) the uncertainties in regional predictions of the magnitude and the direction of climate changes in boreal regions (Roy et al., 2014; Aitken and Bemmels, 2015); (iii) the predicted increase in the occurrence of extreme events and temporal variability in temperature and rainfall under climate change (Bourque and Simonet, 2007); (iv) the possible contribution of non-climatic factors (nutrient availability, soil properties, and mycorrhizal communities) to local adaptation of tree populations (Aitken and Whitlock, 2013; Kranabetter et al., 2015; Pickles et al., 2015); and (v) the possibility that phenotypic plasticity may vary among populations, with populations exhibiting higher levels of plasticity showing likely a greater survival rate but a lower growth rate.

The assessment of phenotypic variation and adaptive value of functional traits involved in carbon assimilation and resource use efficiencies among seed sources in response to variation in climatic conditions should contribute toward more informed ecophysiological-based methods of matching populations to site conditions under climate change (Gray and Hamann, 2013; Isaac-Renton et al., 2014; Prober et al., 2015). The assessment of such traits should enable to improve APM beyond summary parameters such as growth and yield estimates and thereby, help minimize the risk of establishment failure, and maximize the adaptation potential of transferred seed sources.

Variation in functional traits along a climatic gradient is largely influenced by local adaptation and phenotypic plasticity, i.e., the ability of a genotype to produce different phenotypes under different environmental conditions (Valladares et al., 2014; Anderson and Gezon, 2015). Thus, disentangling genetic (local adaptation) and environmental (phenotypic plasticity) responses could improve our understanding and prediction of the physiological and growth responses of populations to climate change (McLean et al., 2014; Merilä and Hendry, 2014; Anderson and Gezon, 2015) and determine the ability of populations to adjust to novel environmental conditions during and after assisted migration (Isaac-Renton et al., 2014; Anderson and Gezon, 2015).

Photosynthetic-related traits are key adaptive and fitness-related functional traits controlling carbon uptake, resources use efficiencies, and relative growth rates. Many studies conducted on different species have recorded among populations phenotypic differentiation for these traits. For instance, leaf nitrogen concentration (*N*_mass_) and respiration rate (*R*_*d*_) have been found to increase with latitude and elevation while specific leaf area (SLA) decreases (Friend et al., 1989; Oleksyn et al., 1998; Hultine and Marshall, 2000; Qiuhong et al., 2013). Photosynthetic rate (*A*_max_) has also been reported to increase with latitude and elevation (Cordell et al., 1998; Oleksyn et al., 1998; Soolanayakanahally et al., 2009), but has been also reported to decrease or to have no linear relationships with latitude or elevation (Kogami et al., 2001; Dang et al., 2008; Qiuhong et al., 2013; Benomar et al., 2015). *A*_max_ is affected by biochemical and biophysical processes and environmental conditions (Sharkey et al., 2007). Indeed, the trend of *A*_max_ along latitude or elevation may be species-specific and may result from the relative limitation imposed by each process.

Carbon isotope discrimination occurring during C_3_ photosynthesis (Δ^13^C; i.e., the ratio of the amount of ^13^C to ^12^C isotopes in a sample relative to a standard) is related to the supply and demand of CO_2_ within the needle. Thereby, Δ^13^C is intrinsically determined by biochemical (carboxylation and photorespiration) and biophysical processes (Farquhar et al., 1989; Cernusak et al., 2013). Leaf Δ^13^C, which scales negatively with WUE (Farquhar et al., 1989; Warren and Adams, 2006), was found to vary with stomatal and mesophyll conductance (Kogami et al., 2001; Warren and Adams, 2006; Flexas et al., 2008), leaf morphology (Körner et al., 1991), leaf age (Ethier et al., 2006), leaf nitrogen concentration (Sparks and Ehleringer, 1997), and as well as with latitude and elevation (Körner et al., 1991; Cernusak et al., 2013). Therefore, Δ^13^C may represent an integrated measure of water and carbon economics mediated by the modulation of biophysical and biochemical processes in response to environmental conditions. Little research has been conducted on genetic variation and phenotypic plasticity in Δ^13^C for white spruce.

White spruce (*Picea glauca* [Moench] Voss) is one of the most commercially important tree species in the boreal forest of North America (Beaulieu et al., 2009). Its natural stands of the commercial forest zone in Québec are located between 45° and 50° N latitude and between 57° and 79°W longitude (de Lafontaine et al., 2010). Evidence is accumulating that white spruce populations within this narrow geographical range are differentiated with latitude, and more weakly with longitude (Khalil, 1985; Li et al., 1997; Jaramillo-Correa et al., 2001; Namroud et al., 2008; Rainville et al., 2014). Our recent results (Benomar et al., 2015; Villeneuve, 2015), under controlled conditions, showed the presence of clinal variation in photosynthetic-related traits. The relative contribution of both phenotypic plasticity and genetic variation to this phenotypic divergence has fundamental implications to improve our understanding and prediction of the physiological and growth responses of white spruce populations to climate change. However, variation in functional trait plasticity among white spruce seed sources remains poorly studied. To our knowledge, this paper is the first to investigate the possible occurrence of clinal patterns of phenotypic plasticity of physiological traits in white spruce. Consequently, the aim of the present study was to disentangle the contributions of local genetic adaptation and physiological plasticity to the observed geographical variation in photosynthetic-related traits among white spruce seed sources during the establishment phase after outplanting under different sites conditions along a regional climatic gradient. We address the following questions: (1) Do photosynthetic-related traits variation in young white spruce reflect local adaptation at least in part? (2) Does plasticity in photosynthetic-related traits in response to climate transfer vary among seed sources? If so, (3) does the degree of plasticity correlate with the climate of seed origin?

## Materials and methods

### Genetic material and seedling production

**Figure 1:**
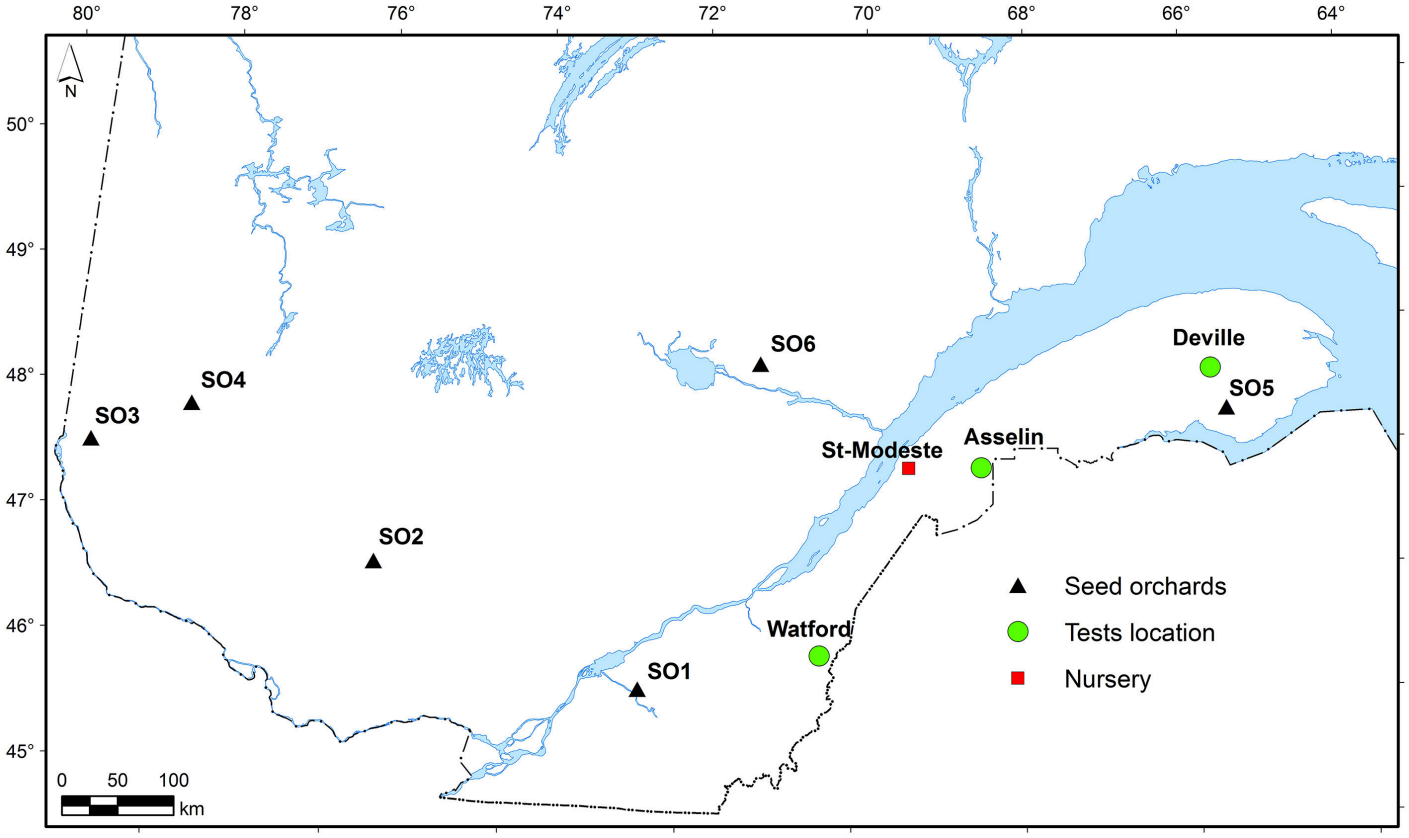
**Location of the planting sites and the six seed sources (SO) used in the current study**.

The white spruce seed sources used in this study came from six first-generation local seed orchards commonly used for reforestation in Québec, Canada (Table 1). The first-generation seed orchards were established about 30 years ago separately for each region using phenotypically selected plus-trees from local natural forests (Figure 1). Open-pollinated seed was collected in each seed orchard for two consecutive years (2008 and 2009). After mixing the collection years for each individual seed orchard, seedlings were produced in a commercial forest nursery at St-Modeste Québec, Canada (47.50°N, 69.23°W) under standard nursery cultural practices in Quebec (Lamhamedi et al., 2006). Seeds having similar seed size among seed sources (seed orchards) were stratified and were manually sown in May 2011 into containers filled with peat:vermiculite (3/1, v/v; bulk density of 0.10 g cm^−3^). Seedlings were cultivated under an unheated polyethylene tunnel during their first growing season and outdoors during their second growing season. Details relating to usual irrigation and fertilization practices during seedlings production in Québec nursery are described in Lamhamedi et al. (2006).

**Table 1:** **Location and climatic conditions^*a*^ (over 1981–2010) of the six white spruce seed sources used in this study and the three plantation sites**.

	**Location**	**LAT**	**LON**	**ELV**	**GDD5**	**MAT**	**TAP**	**MGST**	**TGSP**
		**(°N)**	**(°W)**	**(m)**		**(°C)**	**(mm)**	**(°C)**	**(mm)**
**SEED ORCHARD**									
SO1	Wendover	46.39	71.94	116	1708	4.16	1093	14.85	539
SO2	Fontbrune	46.43	75.74	354	1588	3.42	989	14.41	484
SO3	Baby	47.75	78.47	321	1480	2.03	920	14.16	454
SO4	Desroberts	48.76	77.86	314	1332	0.84	889	13.97	420
SO5	Robidoux	48.55	65.59	270	1298	2.60	1117	12.87	500
SO6	Falardeau	48.54	71.73	351	1347	1.37	1039	13.85	478
PLANTATION SITE^*^									
Watford	Ste-Rose de Watford	46.30	70.40	385	1652	3.70	1185	15.60	468
Asselin	Squatec	47.84	68.52	370	1504	2.40	1042	13.90	412
Deville	Robidoux	48.62	65.72	530	1205	1.50	1210	12.95	310

a *LAT, latitude; LON, longitude; ELV, elevation; GDD5, Number of growing degree-days ≥5°C; MAT, mean annual temperature; TAP, total annual precipitation; MGST, mean growing season temperature; TGSP, total growing season precipitation*.

* *Data for plantation sites are means of years 2013 and 2014*.

### Plantation sites and experimental design

The study area was located in the eastern Canadian boreal forest. Three forest sites were selected for this study and the plantations were established in the spring of 2013: Watford, Asselin, and Deville (Table 1, Figure 1). These sites represent the south, center, and north of the white spruce commercial zone in the eastern part of the boreal forest of Québec, Canada (Figure 1). The Watford (southern) site was formerly occupied by a plantation of black spruce (*Picea mariana* B.S.P. Voss) which was harvested in 2012. It is located in the sugar maple-yellow birch domain (near the locality of Ste-Rose de Watford, Québec, Canada) on a loamy soil. The Asselin (central) site was a former mixed forest stand growing on a loamy soil, which was commercially harvested in 2011. It is located in the balsam fir-yellow birch domain. The Deville (northern) site with clay loamy soil was also dominated by a mixed forest and was commercially harvested in 2012. It is located in the balsam fir-white birch domain. Soil chemical properties before planting at the three sites are given in Table S1.

Two-year-old seedlings were planted at densities of 2000 stems/ha during the last week of May 2013 at Watford, the first week of June at Asselin, and the second week of June at Deville. Before plantation, 15 seedlings per seed source (SO) were used for a quality check, based upon 27 morphological and nutritional standards developed for large containerized white spruce seedlings in Québec (Table S2). Seedling height and root collar diameter (mean ± standard deviation) were 38.3 ± 3.6 cm and 6.9 ± 0.9 mm, respectively. During the first (2013) and the second (2014) growing seasons, weed competition was controlled mechanically at Watford and Asselin, while no treatment was necessary at Deville due to the very limited presence of weedy vegetation.

At each site, we chose a randomized complete block design with four blocks each one was partitioned into six plots within which the six seed sources (SO) were assigned randomly. The size of each plot was about 730 m^2^ and contained 144 trees (12 × 12 rows of trees) in which the 64 interior trees were considered, leaving 4 × 4 rows of border trees as a buffer zone.

### Climate data during the sampling year

**Figure 2:**
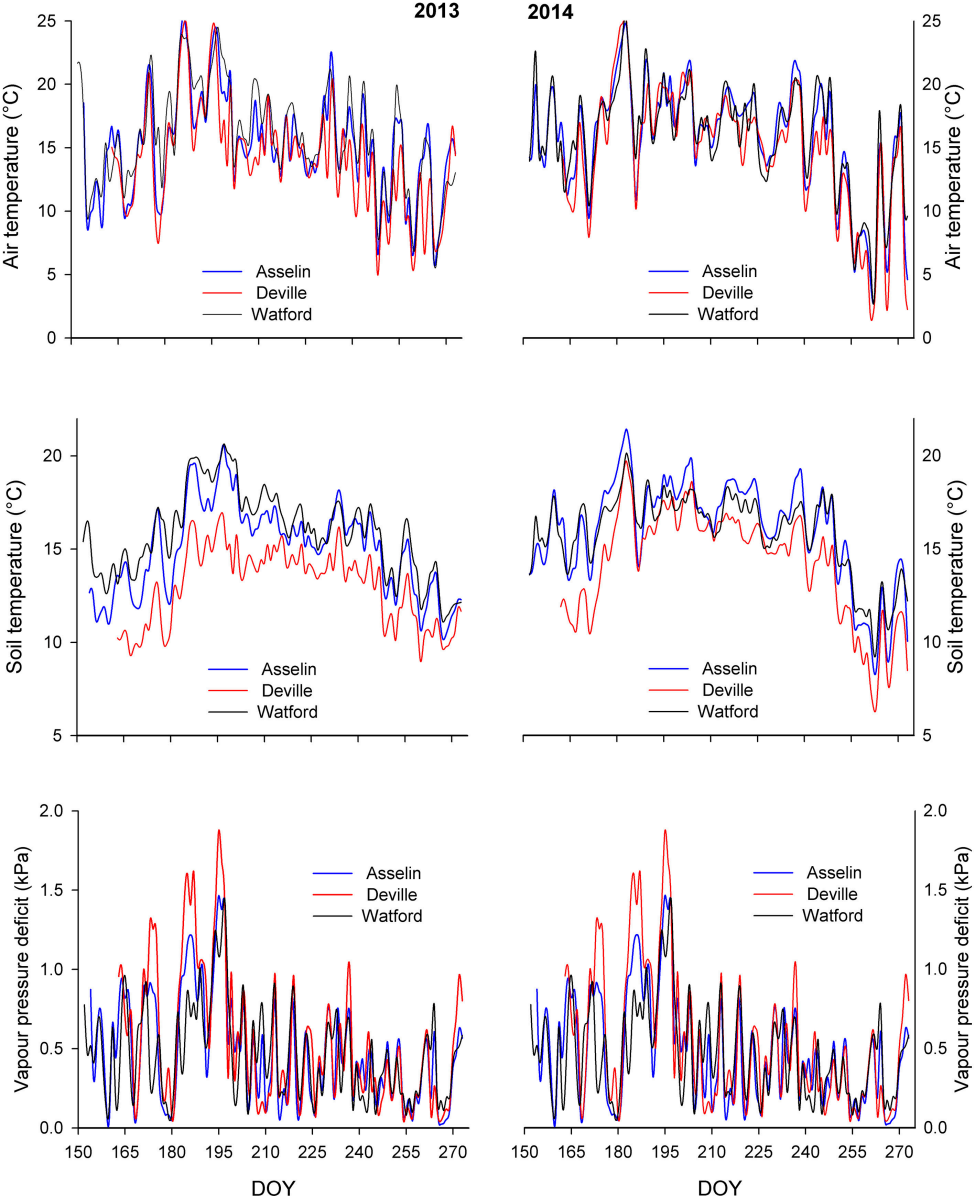
**Mean daily air temperature, mean daily soil temperature at a depth of 10 cm, and mean daily air vapor pressure deficit (VPD) at the three plantation sites during the growing season of 2013 and 2014**. DOY, day of year.

Each site was equipped with a meteorological station. The station was supplied with a shielded air temperature and relative humidity sensor (HMP50, Vaisala, Helsinki, Finland), PAR sensors (Li 190 Campbell Scientific, Logan, UT, USA), pluviometer, and three soil temperature sensors placed at three soil depth (10, 20, and 30 cm). Data were continuously recorded hourly throughout the growing season of 2013 and 2014 (from 152 to 273 day of year) using datalogger (CR10X, Campbell Scientific, Logan, UT, USA) (Figure 2).

### Growth

Height and survival were measured at the end of the second growing season (mid-October 2014) on the 64 central trees in each plot. A total of 4608 seedlings were measured (64 trees ^*^ 4 blocks ^*^ 6 seed sources ^*^ 3 sites). Tree height was measured by a standard 1 m-ruler with 0.5 cm precision.

### Gas exchange measurements

Needle-level gas exchange measurements were made with a portable open-path gas-exchange system (Li-6400, Li-Cor Inc., Lincoln NE), equipped with a Lighted Conifer Chamber (model 6400-22L, Li-Cor Inc., Lincoln NE). Measurements were carried out on one randomly selected plant per plot (6 ^*^ 4 ^*^ 3 = 72 plants). Dark respiration (*R*_*d*_) and photosynthetic carbon dioxide response curves (*A*–*C*_*i*_) were measured on 1-year-old needles within the uppermost lateral shoot. Dark respiration (net CO_2_ exchange at PAR = 0 μmol·m^−2^·s^−1^) was measured before *A*–*C*_*i*_ curve to avoid any influence of high level of CO_2_ on respiration. *R*_*d*_ was recorded after at least 12 min of dark inside the cuvette by turning off LED light source. The *A*–*C*_*i*_ curve measurements were taken after 20 min of steady state conditions at an ambient atmospheric CO_2_ partial pressure (*C*_*a*_ = 400 μmol mol^−1^) and at saturated photosynthetic active radiation (PAR = 1000 μmol m^−2^ s^−1^). Thereafter, the reference CO_2_ (*C*_*a*_) was changed in the following order: *C*_*a*_: 400, 300, 200, 100, 50, 400, 500, 600, 800, 1000, 1200, 1400, and 1500 μmol mol^−1^. Values were recorded based on the stability of photosynthesis, stomatal conductance, CO_2_, and water vapor concentrations. This was generally achieved after 5–7 min at each step. The attachment points of the shoot to cuvette walls were taped (adhesive tape) allowing for, at most, insignificant leaks into and out of the cuvette. Data were not corrected for a diffusion of CO_2_. During measurement, the leaf chamber conditions were: *T* = 25°C, RH = 60 ± 5, VPD = 1.2 ± 0.2 kPa, flow = 300 μmol s^−1^, and PAR = 1000 μmol m^−2^ s^−1^. A total of 72 *A*–*C*_*i*_ curves were measured. The VPD was easily maintained between 1.1 and 1.4 kPa for days with temperature lower than 25°C and problematic to maintain in days with temperature superior to 25°C. Measurements were made from 08h00 to 12h00 and from 14h00 to 17h00 when VPD within the cuvette still within a suitable range (1.0–1.4 kPa).

### Estimation of *V*_*c*max_, *J*_max_, and *g*_*m*_ by curve-fitting method

Photosynthetic parameters such as maximum rates of carboxylation (*V*_*c*max_), maximum rate of electron transport (*J*_max_), and mesophyll conductance (*g*_*m*_: CO_2_ transfer conductance from the intercellular space to the site of carboxylation) were derived from the *A*–*C*_*i*_ response curves. Following Ethier et al. (2006) and Miao et al. (2009), *A*–*C*_*i*_ curves were fit with the biochemical model of C_3_ photosynthesis developed by Farquhar et al. (1980) and modified by Sharkey et al. (2007) using non-linear regression techniques (Proc NLIN, SAS) as described below. The biochemical model of C_3_ leaf photosynthesis assumes that the rate of CO_2_ uptake (*A*_*n*_) is limited by: (i) the Rubisco activity (*A*_*c*_), (ii) the rate of regeneration of the RuBP (*A*_*j*_) and (iii) the rate of use of the triose-P (*A*_*p*_). The final rate, *A*_*n*_, is:

(1) $$A_{n}=min\left(A_{c},A_{j},A_{p}\right)-R_{l}.$$

In the present study, *A*_*p*_ was not considered since it has been rarely observed *in vivo* (von Caemmerer, 2000). *R*_*l*_, respiration occurring in daylight which is assumed to be primarily mitochondrial respiration (von Caemmerer, 2000) and was assumed to approximate dark respiration (*R*_*d*_).

The Rubisco-limited rate of CO_2_ assimilation (*A*_*c*_) is given by:

(2) $$A_{c}=V_{cmax}\;\frac{\left(C_{c}-\;\Gamma^{*}\right)}{C_{c}+K_{c}\left(1+\left[O\right]/K_{o}\right)}$$

where *V*_*c*max_ is the maximum rate of carboxylation; *C*_*c*_, the chloroplastic concentration of CO_2_; Γ^*^, the CO_2_ compensation point and *K*_*c*_ and *K*_*o*_, the Michaelis–Menten constants of Rubisco for CO_2_ and O_2_, respectively. The values used for *K*_*c*_ and *K*_*o*_ were 272 and 165 μmol mo1^−1^, respectively (Sharkey et al., 2007).

The RuBP-limited rate of CO_2_ assimilation (*A*_*j*_) is given by:

(3) $$A_{j}=J\;\frac{C_{c}-I^{*}}{4\left(C_{c}+2I^{*}\right)}$$

where *J* is the rate of electron transport. The rate of electron transport is given by:

(4) $$J=\frac{\alpha \;Q}{\sqrt{1+{\left(\frac{\alpha Q}{J_{max}}\right)}^{2}}}$$

where *J*_max_ is the maximum rate of electron transport; *Q*, the incident PAR; and α, the quantum efficiency which represents the initial slope of the photosynthetic light response curve. Therefore, α is a measure of the efficiency of light absorption by the leaf.

The mesophyll conductance (*g*_*m*_) is given by von Caemmerer (2000):

(5) $$C_{C}=C_{i}-A_{n}/g_{m}$$

where *C*_*i*_, the intercellular concentration of CO_2_.

The mesophyll to stomatal conductance ratio (*g*_*m*_/*g*_*s*_ ratio) was calculated as *g*_*m*_ divided by *g*_*s*_-value at the ambient atmospheric CO_2_ partial pressure (*C*_*a*_ = 400 μmol mol^−1^). Because *g*_*m*_ and *g*_*s*_ may vary partially independently, *g*_*m*_/*g*_*s*_ ratio is increasingly recognized as a useful trait to improve WUE.

### Needle nitrogen concentrations, SLA, and carbon isotope discrimination

Following gas exchange measurements (the *A*/*C*_*i*_ curves), the shoots were carefully removed from the cuvette, and were immediately harvested, placed in plastic bags, and refrigerated (–20°C). Afterward, the needles were scanned and projected leaf area was measured using WinSeedle (Version 2007 Pro, Regent Instruments, Québec, Canada). After oven-drying (56°C for 72 h) and weighting, SLA was calculated as the ratio of the projected needles area (cm^2^) to the needles dry mass (g). Dried needles were ground to a fine powder in a ball mill. Needle nitrogen concentrations (*N*_mass_: mg g^−1^) were determined by high-temperature combustion using a LECO elemental analyzer (Leco Corporation, St-Joseph, Michigan). Nitrogen concentration (*N*_mass_) was then converted to a projected area basis (*N*_*a*_) using the SLA measurements for each sample. Photosynthetic nitrogen-use efficiency (PNUE) was calculated as *A*_max_ divided by *N*_*a*_ (*A*_max_/*N*_*a*_).

Carbon isotope discrimination analysis was performed on needles from the adjacent shoot at the same position in the plant used for gas exchange. After projected leaf area, weighing measurement and grinding, samples were sent to the University of California, Davis, Stable Isotope Facility. The samples were combusted in an online continuous flow dual analyser coupled to an isotope ratio mass spectrometer (Europa Scientific Integra, Cheshire, England, UK). The ratio of ^13^C to ^12^C was expressed as delta (δ^13^)-values in parts per million (‰) with respect to the Vienna Peedee Belemnite (VPDB) carbonate standard following Farquhar et al. (1982):

(6) δ13C=[(13C12C)sample− (13C12C)VPDB](13C12C)VPDB103

where (^13^C/^12^C)_sample_ and (^13^C/^12^C)_VPDB_ are the ratios of ^13^C/^12^C in the sample and the arbitrary standard (V-PDB, Vienna Peedee belemnite), respectively.

The accuracy of the δ^13^C analysis was about 0.05‰, as tested against four standards with known isotopic composition.

The discrimination against ^13^C (Δ^13^C) was calculated following (Farquhar et al., 1989):

(7) $${△}^{13}\text{C}=\frac{\left(\delta^{13}C_{air}-\delta^{13}C_{needle}\right)}{\left(1+\;\delta^{13}C_{needle}\right)}$$

where Δ^13^C is an index of discrimination between the two stable carbon isotopes which is generally used as an indicator of long-term water use efficiency (WUE). Theoretically, Δ^13^C is expected to scale negatively with WUE (Farquhar et al., 1989). δ^13^C_air_ is the carbon isotope ratio of atmospheric CO_2_ (−8‰).

### Climatic data

Climatic data collected from adjacent meteorological stations for the period 1981–2010 were interpolated for each seed source (seed orchard) using the BioSIM software (Régnière and St-Amant, 2007). Latitude, longitude, and altitude of the centroid of plus-trees was used to simulate climatic data of each seed orchard (Table 1). This data set contains biologically relevant climatic indices derived from monthly temperature and precipitation, such as mean growing season temperature (MGST), total growing season precipitation (TGSP), mean annual temperature (MAT), total annual precipitation (TAP), number of growing degree-days ≥5°C (GDD5; Table 1), mean July temperature (MJT), frost-free days (*T*_min_ > 0), total radiation during growing season, and an aridity index. Climates of seed origins (seed orchards) showed latitudinal and longitudinal gradients. Although temperature and precipitation varied with latitude and longitude, precipitation was strongly correlated to longitude of seed origin, while MGST, mean July temperature (MJT) and number of growing degree days ≥5°C (GDD5) were strongly correlated to latitude (Benomar et al., 2015).

### Statistical analyses

All analyses were conducted with SAS version 9.4 (SAS Institute, Cary, NC, USA). Except for survival, response variables were analyzed separately by the MIXED procedure using a general linear mixed model with the effects of site and seed source (seed orchard) and their interaction on response variables as fixed effects and block as random effect. A significant seed source effect means that there is genetic differentiation among seed orchards, while a significant site effect is synonymous with trait plasticity and a significant site ^*^ seed source interaction effect means that plasticity varies among seed sources. Data were transformed when required to satisfy normality of residuals and homoscedasticity. The proportion of variance explained by each factor (site, seed source, and site ^*^ seed source) was estimated by PROC VARCOMP. Means comparisons were performed using the Tukey test; differences were considered significant at *P* = 0.05. Survival rates of seedlings at the end of the second growing season in the three sites were compared using the χ^2^-test (PROC FREQ). When ANOVAs showed a significant effect of seed source for a trait, the genetic differentiation along the climatic gradient was tested by univariate linear regression of that trait (mean values across sites) and climate of seed origin (MAP, GDDD, and MJT) using PROC REG (SAS Institute, Cary, NC, USA). The linear regressions were considered significant when *P* < 0.05 and marginally significant when *P* < 0.1. When ANOVAS showed a significant effect of the interaction between site and seed source, clinal variation of plasticity along the climatic gradient was tested by univariate linear regression of means of seed sources at each site for a given trait on climate of seed origin.

The phenotypic plasticity was assessed quantitatively for each trait using the relative distance plasticity index (RDPI) as described by Valladares et al. (2007):

(8) $$RDPI=\sum \frac{\left|mean_{ij}-\;mean_{\overset{`}{i}\overset{`}{j}}\right|}{\left(mean_{ij}+mean_{\overset{´}{i}\overset{´}{j}}\right)}/n$$

where mean_*ij*_ is the mean value of trait of seed source *i* (*i* = 1, … ., 6) growing at site *j* (*j* = 1, … , 3) and *n* is the total number of distances (*n* = 3). For each trait, one value of RDPI was calculated for each seed source across treatments which were considered as replicates. This allows statistical comparison of RDPI between traits using simple ANOVAs. As RDPI ranges from 0 (no plasticity) to 1 (maximal plasticity), data were transformed using logit transformation prior to the analysis. An analysis of covariance (ANCOVA) was used to assess the effect of site on the slope and intercept of the relationships between height (H2014) and functional traits.

## Results

### Seedling performance

**Figure 3:**
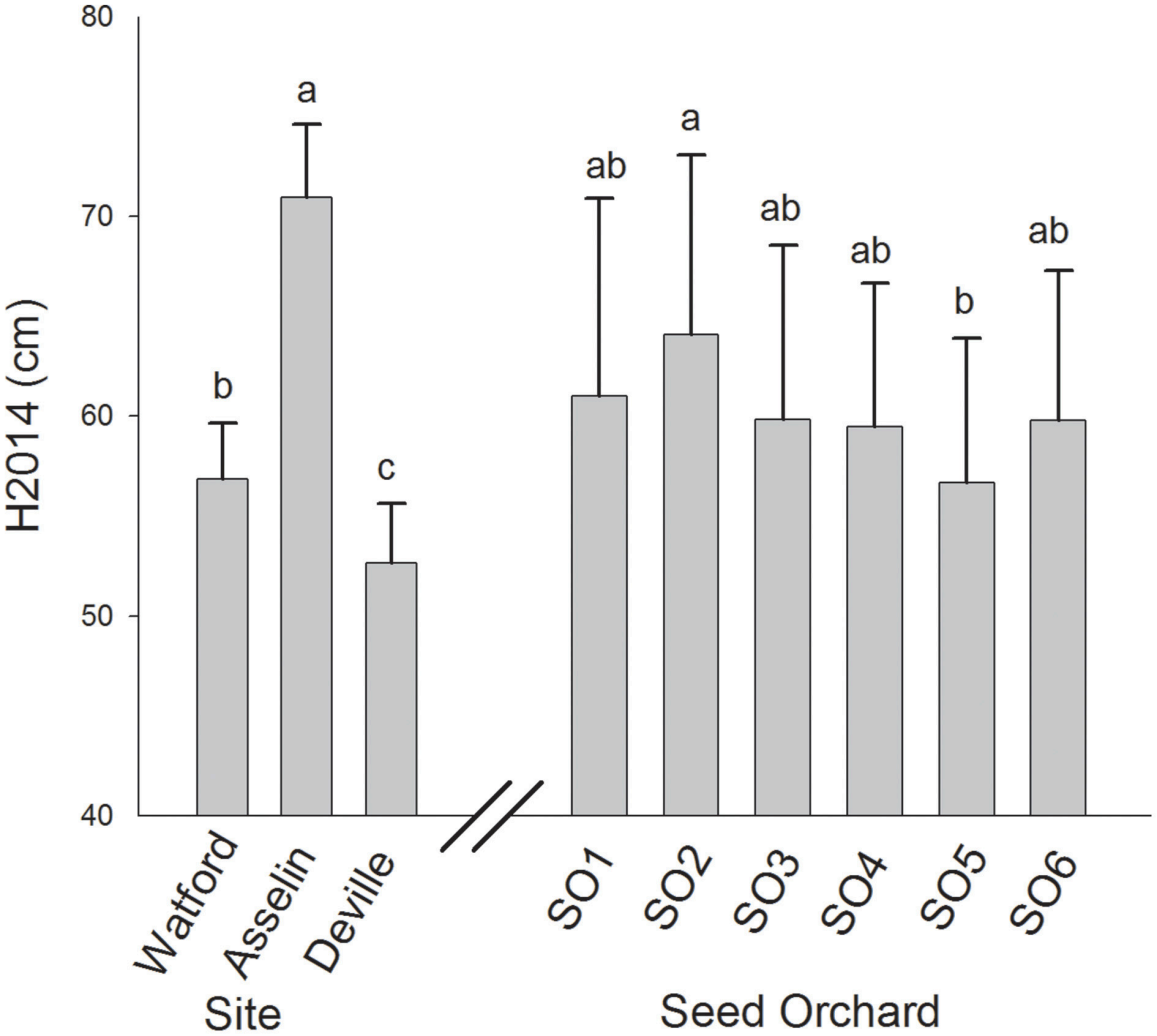
**Mean total height growth at the end of the second growing season (H2014) of white spruce seedlings from six seed orchards at three plantation sites**. Means having the same letters are not significantly different at α = 0.05. Values are the means ± SD.

**Figure 4:**
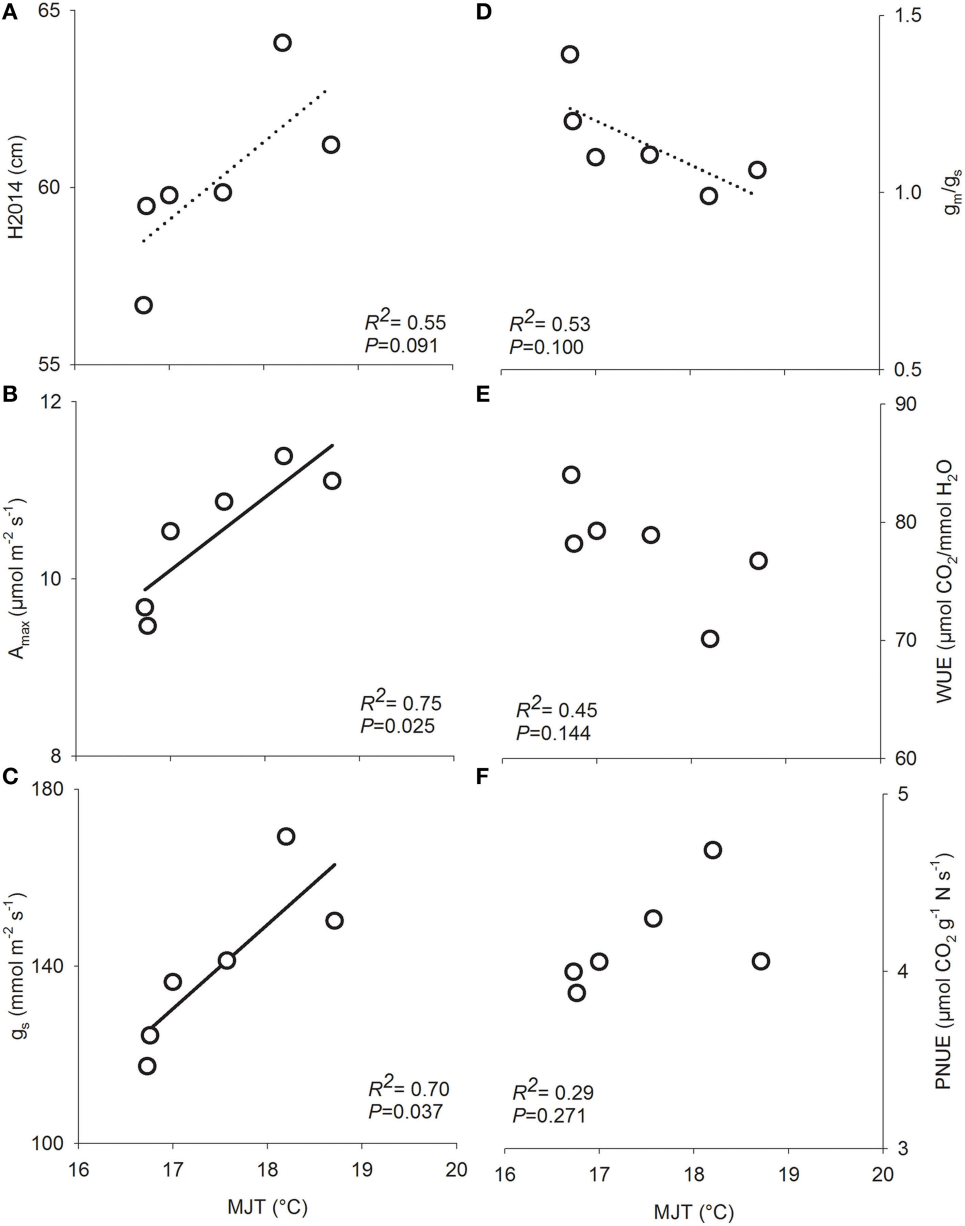
**Relationships between functional traits in six white spruce seed sources and the mean July temperature at their origin (MJT) after growing on three forest sites**. **(A)** Total height at the end of the second growing season (H2014), **(B)** light-saturated photosynthesis on a projected leaf area basis (*A*_max_), **(C)** stomatal conductance (*g*_*s*_), **(D)** mesophyll to stomatal conductance ratio (*g*_*m*_/*g*_*s*_), **(E)** water use efficiency (WUE), and **(F)** photosynthetic nitrogen-use efficiency (PNUE). Data points are means of 12 seedlings for functional traits and 768 seedlings for growth (H2014). Regression lines and coefficient of determination (*R*^2^) values are shown for significant relationships, with solid line for *P* < 0.05 and dashed lines for *P* < 0.1 (*n* = 6).

Total height growth after two growing seasons (H2014) was significantly affected by both site and seed source. However, the interaction between site and seed source was not significant, indicating that the plasticity of seedlings from the six seed sources was similar in response to changes in site conditions along the climatic gradient (Table 2). Variance components showed that, on average, the plantation site effect explained the largest portion of variance (75%) while the seed source effect explained only 9% of the observed variance. Height growth was the greatest in Asselin (central) followed by Watford (south), and Deville (north), respectively (Figure 3). The seedlings from SO2 were significantly taller than those from SO5, and seedlings from other seed orchards (SO1, SO3, SO4, and SO6) had intermediate height values (Figure 3). There was a significant clinal variation among seed sources in height growth; in fact, height growth was negatively associated with latitude of seed origin (*P* < 0.06; *R*^2^ = 0.63) and marginally (*P* = 0.1) and positively associated with MJT (Figure 4A). Survival rate was similar among sites and seed sources, and it reached 97% at the end of the second growing season.

**Table 2:** Analysis of variance for growth and functional traits for sources of variation (sites, seed sources and the site ^*^ seed source interaction), degrees of freedom (*df*), *F*-values, and associated probabilities (*P*).

**Traits^a^**	**Sites**		**Seed sources**		Site ^*^ seed source	
	**(*df* = 2)**		**(*df* = 5)**		**(*df* = 10)**	
	***F***	***P***	***F***	***P***	***F***	***P***
H2014	606.50	< 0.001	19.14	< 0.001	1.56	0.14
*A*_max∕area_	16.76	< 0.001	3.49	0.01	0.81	0.62
*A*_max∕mass_	11.67	0.01	2.76	0.03	0.55	0.84
*R*_*d*_	5.22	0.01	0.92	0.47	0.48	0.89
*V*_*c*max_	9.16	< 0.001	0.79	0.56	1.1	0.38
*J*_max_	9.63	0.0004	0.4	0.84	1.11	0.37
*g*_*s*_	19.74	0.001	4.05	0.003	0.86	0.57
*g*_*m*_	5.46	0.008	1.22	0.32	1.04	0.43
*g*_*m*_/*g*_*s*_	5.80	< 0.001	2.17	0.07	1.71	0.11
*N*_mass_	25.02	< 0.001	0.84	0.52	1.07	0.39
SLA	10.46	0.001	1.28	0.28	0.86	0.58
WUE	10.62	< 0.001	3.26	0.008	1.16	0.33
PNUE	14.44	< 0.001	2.95	0.021	1.58	0.14
Δ^13^C	33.16	< 0.001	0.56	0.73	0.88	0.56

^a^Trait abbreviations and symbols: H2014, total height at the end of the second growing season; A_max∕area_, light-saturated photosynthesis on a projected leaf area basis; A_max∕mass_, light-saturated photosynthesis on mass basis; R_d_, dark respiration; V_cmax_, maximum rate of Rubisco carboxylation; J_max_, maximum rate of photosynthetic electron transport; g_s_, stomatal conductance; g_m_, mesophyll conductance; g_m_/g_s_, mesophyll to stomatal conductance ratio; N_mass_, needle nitrogen concentration; SLA, specific leaf area; WUE, water use efficiency; PNUE, photosynthetic nitrogen-use efficiency; Δ^13^C, Needle carbon isotope ratio.

### Functional traits: local adaptation vs. plasticity

All measured ecophysiological traits were under the influence of both site (plasticity) and seed source (genetic differentiation) effects. However, and similarly as for height growth, the interaction between site and seed source was not significant for any of the measured traits, indicating a similar plasticity of traits between seed sources in response to the climatic gradient (Table 2). The percent of variance explained by the site effect ranged from 43 to 75% among measured traits.

Light-saturated photosynthetic rate (*A*_max_) expressed on both a projected area (*A*_max∕area_) and a mass basis (*A*_max∕mass_) showed plasticity in response to changes in growing conditions along the climatic gradient (Table 3). *A*_max∕area_ and *A*_max∕mass_ were greater at Asselin (central) than Watford (south) and Deville (north) (Table 3). There was a significant effect of seed source on *A*_max_ (Table 2). Variation in light-saturated photosynthetic rate on a projected area (*A*_max∕area_) was negatively associated with latitude of seed origin (*P* < 0.05; *R*^2^ = 0.75) and positively with MJT (Figure 4B). Stomatal and mesophyll conductances (*g*_*s*_ and *g*_*m*_) were significantly impacted by site conditions. However, only *g*_*s*_ was significantly influenced by seed source (Table 2). Stomatal conductance was greater at Asselin (central) followed by Watford (south), and Deville (north), respectively. Mesophyll conductance was the greatest in Asselin (central; Table 3), while the mesophyll to stomatal conductance ratio (*g*_*m*_/*g*_*s*_ ratio) was the greatest in Deville (north; Table 3). As for *A*_max_, *g*_*s*_ was negatively associated with latitude of seed origin (*P* < 0.05; *R*^2^ = 0.78) and positively with MJT (Figure 4C). *g*_*m*_/*g*_*s*_ ratio was marginally negatively associated with MJT (Figure 4D). Biochemical limitations to photosynthesis (*V*_*c*max_ and *J*_max_) were significantly affected by site, but not by the seed source (Table 2). *V*_*c*max_ and *J*_max_ were the lowest at Watford (south; Table 3). We found a significant effect of site, but not of the seed source, on SLA and needle nitrogen content (*N*_mass_) (Table 3). *N*_mass_ was the lowest (30%) in Watford (south) and SLA was the greatest in Deville (north; Table 3).

**Table 3:** **Differences in functional traits among the three plantation sites^1^**.

**Traits^2^**	**Sites**		
	**Watford**	**Asselin**	**Deville**
*A*_max∕area_ (μmol CO_2_ m^−2^ s^−1^)	10.0(1.6)b	11.8(1.6)a	9.7(1.5)b
*A*_max∕mass_ (nmol CO_2_ g^−1^ s^−1^)	38.4(7.9)c	47.6(7.1)a	42.7(6.9)b
*R*_*d*_ (μmol CO_2_ m^−2^ s^−1^)	–2.1b(0.5)	–2.6(0.6)a	–2.4(0.5)a
*V*_*c*max_ (μmol CO_2_ m^−2^ s^−1^)	58(9)b	66(7)a	65(8)a
*J*_max_ (μmol CO_2_ m^−2^ s^−1^)	112(16)b	128(15)a	125(13)a
*g*_*s*_ (mmol m^−2^ s^−1^)	138(33)b	169(36)a	112(25)c
*g*_*m*_ (mmol m^−2^ s^−1^)	140(12)b	174(10)a	141(10)b
*g*_*m*_/*g*_*s*_	1.06(0.3)b	1.02(0.2)b	1.30(0.3)a
*N*_mass_(mg g^−1^)	8.7(1.5)b	11.1(1.9)a	11.6(1.7)a
SLA (cm^2^ g^−1^)	38(3)b	40(3)b	44(4)a
WUE (μmol CO_2_/mol H_2_O)	74(12)b	71(8)b	88(10)a
PNUE (μmol CO_2_ g^−1^ N s^−1^)	4.5(0.8)a	4.3(0.6)a	3.7(0.7)b
Δ^13^C (‰)	22.3(0.6)a	21.6(0.6)b	20.9(0.7)c

1 *Within rows, means followed by the same letter do not differ significantly at α = 0.05 based on Tukey's tests*.

2 *Trait abbreviations and symbols: A_max∕area_, light-saturated photosynthesis on a projected leaf area basis; A_max∕mass_, light-saturated photosynthesis on mass basis; R_d_, dark respiration; V_cmax_, maximum rate of Rubisco carboxylation; J_max_, maximum rate of photosynthetic electron transport; g_s_, stomatal conductance; g_m_, mesophyll conductance; g_m_/g_s_, mesophyll to stomatal conductance ratio; N_mass_, needle nitrogen concentration; SLA, specific leaf area; WUE, water use efficiency; PNUE, photosynthetic nitrogen-use efficiency; Δ^13^C, Needle carbon isotope ratio. Values are the means ± SD*.

Site significantly influenced carbon isotope discrimination (Δ^13^C) with a substantial difference between the three sites but unexpectedly, no difference was detected among seed sources (Table 2). Intrinsic water use efficiency (WUE) and photosynthetic nitrogen-use efficiency (PNUE) were under the influence of site and the seed source (Table 2). Deville (north) showed the greatest WUE and the lowest PNUE-values (Table 3). Despite the presence of significant variation in WUE and PNUE among seed sources, the latter were not associated with latitude of seed origin or associated climatic variables (Figures 4E,F).

### Level of plasticity between functional traits

**Figure 5:**
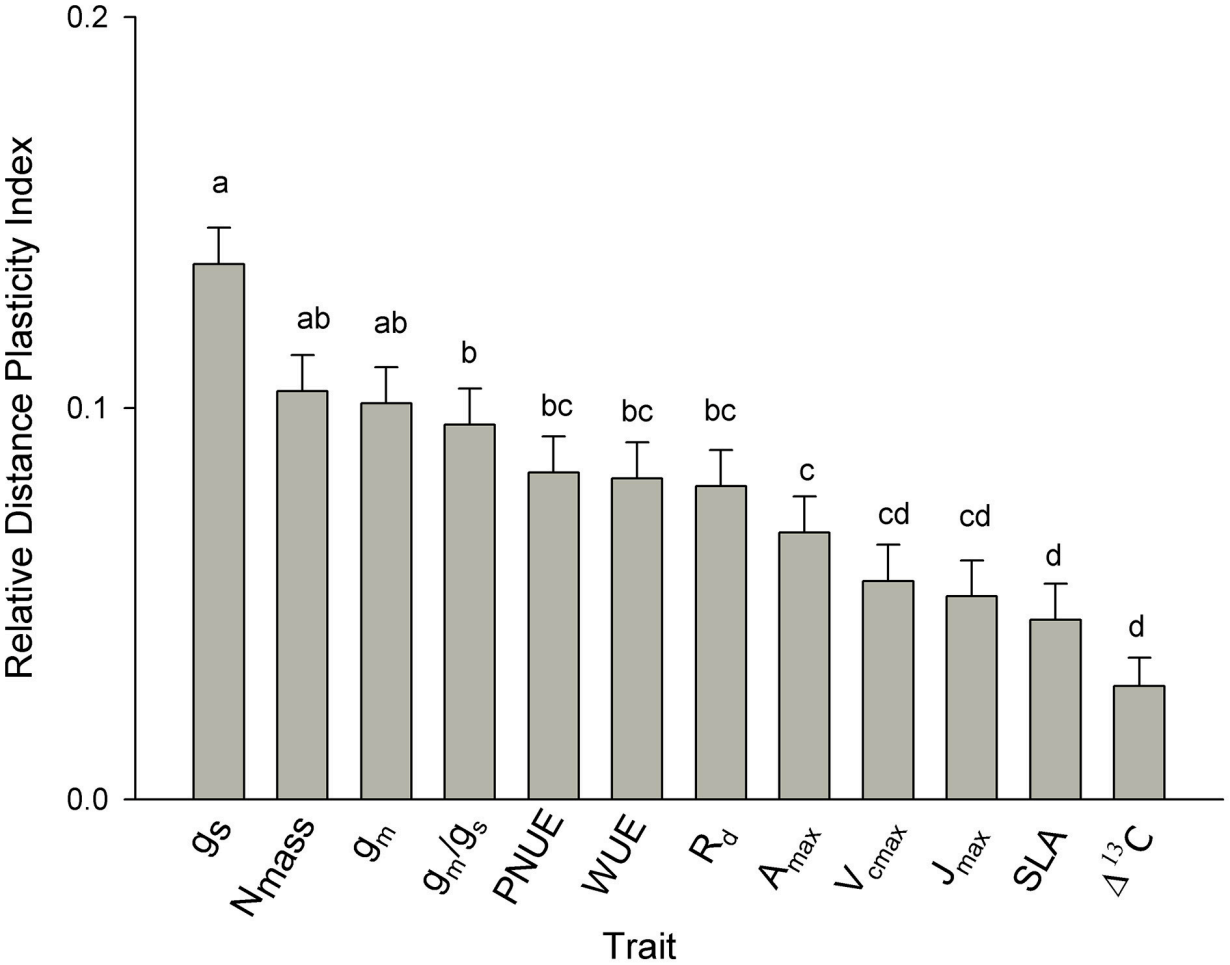
**Comparison of phenotypic plasticity among traits across all seed sources as assessed by relative distance plasticity index (RDPI)**. Means having the same letters are not significantly different at α = 0.05 (Tukey *post hoc*). Values are the means ± SD. (see Table 2 for abbreviations).

As assessed by the relative distance plasticity index (RDPI) across all seed sources, plasticity varied between traits. Values of RDPI ranged from 0.03 to 0.17. Among needle functional traits, stomatal conductance, mesophyll conductance, and *N*_mass_ were the most plastic traits along the climatic gradient with RDPI = 0.14, 0.11, and 0.10, respectively. Needle morphology (SLA), Δ^13^C and biochemical limitation to photosynthesis (*V*_*c*max_ and *J*_max_) were the least plastic traits (Figure 5). All other traits were of intermediate plasticity.

### Phenotypic correlations among traits

Photosynthetic capacity on a projected area (*A*_max∕area_) was positively correlated with stomatal conductance (*g*_*s*_), mesophyll conductance (*g*_*m*_), *g*_*m*_/*g*_*s*_ ratio, needle nitrogen concentration area basis (*N*_area_), and PNUE. On the other hand, *A*_max∕area_ was negatively correlated with *R*_*d*_ and WUE (Table 4). *R*_*d*_ was strongly and positively correlated with *V*_*c*max_, *J*_max_ and needle nitrogen concentrations on both area and mass basis (*N*_area_ and *N*_mass_). Stomatal and mesophyll conductance were positively correlated. Water use efficiency (WUE) was correlated negatively with *g*_*s*_, but not with *g*_*m*_ and it was correlated positively with the *g*_*m*_/*g*_*s*_ ratio (Table 4). PNUE was correlated to *g*_*m*_/*g*_*s*_ ratio, and *N*_mass_. The trade-off between PNUE and WUE was stronger. Δ^13^C was correlated with *R*_*d*_, *g*_*m*_/*g*_*s*_ ratio, *V*_*c*max_, *J*_max_, SLA, WUE, and PNUE (Table 4).

**Table 4:** **Pearson correlations (*r*)^a^ among seed source mean values per plantation site for functional traits (*n* = 18)**.

	***A*_max_**	***R*_*d*_**	***g*_*s*_**	***g*_*m*_**	***g*_*m*_/*g*_*s*_**	***V*_*c*__*max*_**	***J*_*max*_**	***N*_*mass*_**	***N*_*area*_**	**SLA**	**Δ^13^C**	**WUE**
*R*_*d*_	–0.44											
*g*_*s*_	**0.92**	−0.17										
*g*_*m*_	**0.57**	−0.22	**0.49**									
*g*_*m*_/*g*_*s*_	**−0.59**	−0.07	**−0.76**	0.14								
*V*_*c*max_	0.39	**−0.78**	0.14	0.10	0.08							
*J*_max_	0.40	**−0.83**	0.13	0.07	0.06	**0.96**						
*N*_mass_	0.27	**−0.78**	−0.08	0.05	0.26	**0.75**	**0.83**					
*N*_area_	**0.51**	**−0.86**	0.20	0.24	0.13	**0.70**	**0.79**	**0.87**				
SLA	−0.29	−0.16	−0.45	−0.17	0.34	0.30	0.32	**0.51**	0.04			
Δ^13^C	0.03	**0.51**	0.38	0.05	**−0.48**	**−0.68**	**−0.68**	**−0.85**	**−0.64**	**−0.56**		
WUE	**−0.65**	−0.17	**−0.88**	−0.25	**0.83**	0.18	0.23	**0.51**	0.24	**0.53**	**−0.64**	
PNUE	0.46	0.45	**0.65**	0.32	**−0.61**	−0.36	−0.44	**−0.62**	**−0.52**	−0.30	**0.68**	**−0.78**

a *Significant correlations are in bold (P < 0.05), marginally significant correlations are underlined (P < 0.067). Trait abbreviations and symbols are as in Table 3*.

**Figure 6:**
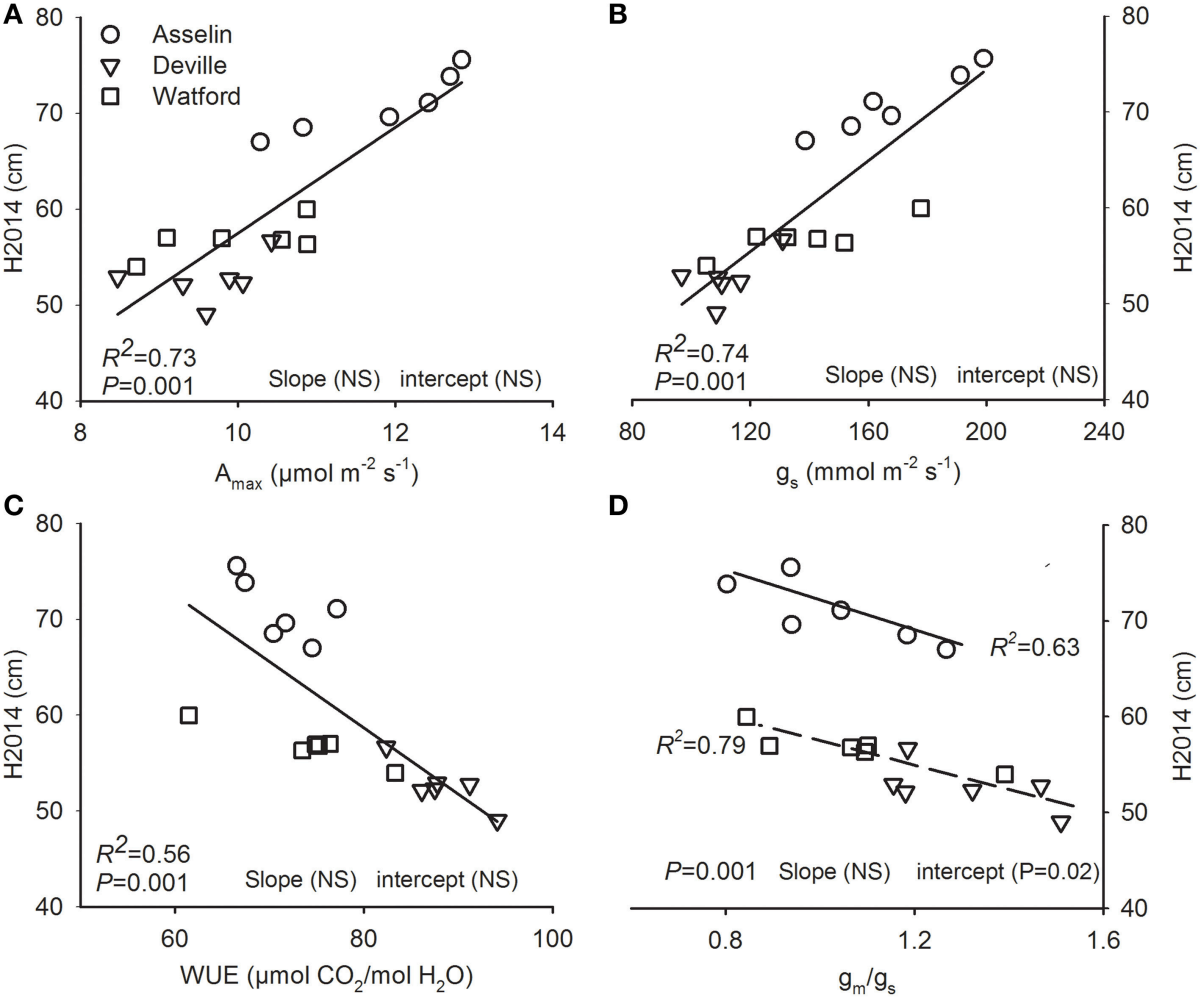
**Total height growth at the end of the second growing season (H2014; an index for fitness) plotted against (A) light-saturated photosynthesis on a projected leaf area basis (*A*_max_) and (B) stomatal conductance (*g*_*s*_), (C) water use efficiency (WUE), (D) mesophyll to stomatal conductance ratio (*g*_*m*_/*g*_*s*_)**. Data points are seed source-specific mean value at each site (*n* = 18). The strength of relationships is indicated by the *p*-value and coefficient of determination. The significant effect of site on slope and intercept of relationships was determined by ANCOVA. Where slope and/or intercept were significantly different, regression line and coefficient of determination are given for each relationships and the *P*-value is given for the common regression. NS, not significant.

Height was positively related to *A*_max∕area_, and *g*_*s*_ and negatively to *g*_*m*_/*g*_*s*_ ratio and WUE. Height–*A*_max∕area_, height–*g*_*s*_, and Height–WUE relationships were insensitive to environment conditions (site of plantation; Figures 6A–C). The intercept was significantly greater in Asselin (central) for the relationship of H2014 with *g*_*m*_/*g*_*s*_ ratio (Figure 6D). H2014 was unrelated to all other measured functional traits (*P* ≥ 0.05).

## Discussion

### Growth and performance of white spruce seedlings from different seed sources under different site conditions

Our study revealed a clinal gradient in height growth at a regional scale among the six tested seed sources (Figure 4A). Irrespective of growing conditions (plantation site), southern seed sources grew faster than northern seed sources (Figure 3). In addition, the survival rate was very high (97%) and similar among seed sources. These results are in agreement with previous reports showing negative latitudinal cline of height growth for young white spruce at regional (Li et al., 1997; Jaramillo-Correa et al., 2001; Thomson et al., 2010; Carles et al., 2011) and range-wide scales (Lu et al., 2014). A negative latitudinal cline in growth rate has also been reported for other boreal conifer species (Matyas and Yeatman, 1992; Wei et al., 2004). Stott and Loehle (1998), suggesting that this pattern may result from the pressure exercised by both biotic and abiotic factors. Populations from the southern range of the species may exhibit a competitive growth strategy as a consequence of higher level of biotic pressures (interspecific competition), while northern populations may exhibit a more conservative growth strategy focused on survival growth strategy as a consequence of higher level of abiotic pressure (cold damage, drought). Our results suggest that the observed trend in height growth with latitude may be driven by the trade-off between *A*_max_ and WUE mediated by genetic variation in *g*_*s*_ and *g*_*m*_/*g*_*s*_ ratio (Figure 6) rather than the trade-off between growth and survival.

The growth of seed sources along the climatic gradient followed a parabolic pattern. Height growth was 30% greater for the site with the intermediate climate (Asselin) followed by the warmest (Watford) and the coldest (Deville) sites, respectively. The lower height growth at the warmest site (Watford) may be interpreted as an existing maladaptation of tested seed sources to warming conditions in southern Québec (Rainville et al., 2014). Considering previous findings showing a strong age-age-genetic correlation of height growth in white spruce (Li et al., 1993; Wahid et al., 2013), one may believe that this observed parabolic growth pattern could be stable over the next years during the juvenile stage. Longer-term data are however required to confirm this pattern, although a similar pattern was observed with older white spruce seed sources on different sites (Andalo et al., 2005).

### Functional trait expression: local adaptation vs. plasticity

We found a significant effect of site and seed source on *A*_max_, *g*_*s*_, *g*_*m*_/*g*_*s*_ ratio, WUE, and PNUE. However, the interaction between site and seed source was not significant suggesting a lack of differential plasticity among seed sources. The low variation explained by seed sources (9–18%) for the above-mentioned traits suggests that genetic differentiation in these traits is limited. Nevertheless, the results indicate that white spruce populations in Québec are locally adapted despite the limited latitudinal range sampled in the present study and the large gene flow existing in this widespread boreal tree species (Jaramillo-Correa et al., 2001). The results of the present study are in agreement with previous findings showing a significant genetic differentiation among populations in the same region for growth, phenology, morphology, and physiology traits (Li et al., 1997; Namroud et al., 2008; Carles et al., 2011; Benomar et al., 2015).

Photosynthetic rate (*A*_max_) showed a clinal variation with southern populations exhibiting a higher *A*_max_ than northern populations (Figure 4B). This pattern is in agreement with previous findings (Kogami et al., 2001; Dang et al., 2008; Qiuhong et al., 2013; Benomar et al., 2015). However, the results are in conflict with others that demonstrated a positive latitudinal cline in *A*_max_ driven by needle nitrogen (*N*_mass_) and carboxylation capacity (*V*_*c*max_) (Oleksyn et al., 1998; Soolanayakanahally et al., 2009). Our results showed no genetic variation in respiration rate (*R*_*d*_), *g*_*m*_, *V*_*c*max_, or *N*_mass_. On the other hand, *A*_max_ was strongly correlated with *g*_*s*_, *g*_*m*_, and *g*_*m*_/*g*_*s*_ ratio, but not with *V*_*c*max_ and *J*_max_. Therefore, the latitudinal cline in *A*_max_ in this study likely resulted from a parallel cline in CO_2_ diffusion (*g*_*s*_ and *g*_*m*_/*g*_*s*_ ratio). Overall, our study revealed that the variation among white spruce seed sources in height growth, *A*_max_ and resources use efficiencies was driven by the physics of CO_2_ diffusion (CO_2_ transfer conductance).

Our results are in agreement with the fact that stomatal conductance (*g*_*s*_) is under both environmental and genetic controls. The decrease in stomatal conductance and the increase in *g*_*m*_/*g*_*s*_ ratio and WUE along with latitude may arise from a water conservation strategy in northern latitude as an adaptation of stomatal characteristics (stomatal density, stomatal length) and hydraulic conductance to a decrease in soil temperature (Wang et al., 2014). In fact, mean soil temperature during the two growing seasons of experiment was 2.4°C lower in the coldest site (Deville). The decrease in soil temperature with latitude would impose an apparent drought stress likely induced by an increase in water viscosity and a decrease in water and nutrient uptake by roots at cold temperatures (Cochard et al., 2000; Luo et al., 2006; Wang et al., 2014). Under boreal forest conditions, low soil temperature has been reported to decrease both stomatal conductance and root growth (Lamhamedi and Bernier, 1994; Grossnickle, 2000; Dang and Cheng, 2004). Thus, the effect of soil temperature on stomatal conductance may arise from combination of poor root growth, low water uptake and plant resistance to water flow (Grossnickle, 1988).

SLA is an adaptive morphological trait determining plant capacity to succeed in specific biophysical environments. Indeed, SLA influences *A*_max_, *R*_d_, *V*_*c*max_ *N*_mass_, and leaf life-span (Wright et al., 2004) and SLA has been reported to be under both genetic and environmental control. A decrease in SLA with increases in latitude and elevation has been reported for several conifer species (Friend et al., 1989; Oleksyn et al., 1998; Hultine and Marshall, 2000; Qiuhong et al., 2013). In the present study, we observed only a small change (14%) in SLA along the climatic gradient. As a result, this trait was less informative regarding phenotypic variation in the physiological traits assessed (Table 4). Changes in SLA may result from changes in needle density and/or thickness. Also, needle density and thickness may change in opposite directions leading to unchanged SLA. Thus, a change in needle morphology may possibly occur but not be captured by SLA. Otherwise, the climatic gradient in our study may likely be insufficient to induce a significant change in SLA.

In agreement with previous reports, we found an environmental influence on carbon isotope composition as demonstrated by the significant differences between sites (Körner et al., 1991; Cernusak et al., 2013; McLean et al., 2014). Δ^13^C increased linearly from the warmest site to the coldest site by 1.4‰ (Kogami et al., 2001). The observed variation in Δ^13^C in this study resulted from a change in *g*_*m*_/*g*_*s*_ ratio, *V*_*c*max_, and *R*_*d*_. The lack of variation in Δ^13^C among seed sources was likely due to the lack of variation between seed sources in *V*_*c*max_ and *R*_*d*_. Our results demonstrate that the change in Δ^13^C is influenced by both biophysical and biochemical processes (Farquhar et al., 1989; Cernusak et al., 2013). The latter may explain the significant correlation between Δ^13^C and both WUE and PNUE.

### Implications for assisted population migration

The southern transfer of northern seed sources led to an enhancement of growth which was still below that of southern seed sources (Figure 3). These results suggest that as temperature warms, growth of northern seed sources of white spruce in Quebec would increase but without reaching that of seed sources already adapted to these new conditions, at least at the young age. In addition, a trade-off between growth and survival did not occur along the tested climatic gradient. Thus, if the objective is to improve forest plantation productivity under climate change, northward assisted migration of southern seed sources (for instance, by 2° of latitude northward) might be possible without undue risk. Furthermore, the faster growth rate of southern seed sources during the juvenile stage may contribute to the establishment success of plantation during assisted migration by avoiding negative impacts of biotic and abiotic factors such as weedy competition, early and late frost damage, and drought stress. The greatest growth rate of southern seed sources seems to have been reached to the detriment of WUE (Figure 6). For this reason and as it was noted by Andalo et al. (2005) that local moisture conditions may also play an important role in white spruce seed source variation, it would be advisable to choose plantation sites based on projected precipitation before any implementation of long distance transfer of white spruce seed sources, as suggested recently by Lu et al. (2014). On the other hand, investigations regarding variation in drought tolerance among seed sources are necessary. In particular, (i) genetic variation and plastic changes in the vulnerability of xylem to cavitation and embolism resistance under water stress, and (ii) the linkage between hydraulic functioning and stomatal conductance should be further studied.

## Conclusion

The present study was motivated by the need to examine the environmental and genetic influences on functional traits variation among white spruce seed sources to assist reforestation decisions under a changing climate. Our study revealed that (i) the seed sources differed in growth, photosynthetic rate (*A*_max_) and its biophysical limitations, and resources use efficiencies; (ii) the seed sources demonstrated similar levels of traits plasticity, suggesting that the observed variation in growth among seed sources resulted largely from genetic variation in functional traits rather than from plasticity. Overall, the results suggest that phenotypic plasticity may allow northern white spruce seed sources to cope over the short-term with a moderate climate change. However, assisted migration under the form of northward seed source transfer from southern areas would still be valuable to increase forest productivity in the northern regions of Québec.

The results of this study were obtained on young material at the juvenile stage. While some short-term conclusions can be drawn with regard to APM, but a long-term follow-up remains necessary to confirm these preliminary findings. Moreover, access to data from additional test sites would help strengthen trends observed. In relation to this, we are in the process of establishing six new tests in order to delineate APM guidelines based on more exhaustive scientific evidence for white spruce in eastern Canada.

## Author contributions

HM, ML, JBo, JBe, AR and LB conceived the study, obtained the funding and participated in the drafting of the manuscript. AR participated to designing the genetic tests and providing the seed. ML and his team have installed three weather stations for the acquisition of environmental variables and conducted mineral analyses. LB performed the measurements of different variables at different sites, the analysis and the interpretation of data. All authors read and approved the final version of the manuscript.

## Funding

This research was funded by grants to HM from the program “Partenariat sur L'aménagement et L'ennvironnement Forestiers” of the Fonds de la Recherche du Québec sur la Nature et les Technologies (FRQ-NT), and the Discovery Grant program of the Natural Sciences and Engineering Research Council of Canada. Major additional support was also provided to ML by the Direction de la Recherche Forestière of the Ministère des Forêts, de la Faune et des Parcs of Québec.

### Conflict of interest statement

The authors declare that the research was conducted in the absence of any commercial or financial relationships that could be construed as a potential conflict of interest.
