# Supplementary material for: Genetic Adaptation vs. Ecophysiological Plasticity of Photosynthetic-Related Traits in Young Picea glauca Trees along a Regional Climatic Gradient
Source: Front Plant Sci. 2016 Feb 3;7:48. doi: 10.3389/fpls.2016.00048 (PMC4737914; doi:10.3389/fpls.2016.00048)
Supplement: Supplementary file 1 [file Table1.DOCX]

***Supplementary Material***

**Genetic adaptation *versus* ecophysiological plasticity of photosynthetic-related traits in young *Picea glauca* trees along a regional climatic gradient**

Lahcen Benomar*, Mohammed S. Lamhamedi, André Rainville, Jean Beaulieu, Jean Bousquet, Hank A. Margolis

*** Correspondence** : Lahcen Benomar : lahcen.benomar.1@ulaval.ca

Supplementary tables

Supplementary Table 1: Selected soil chemical properties collected from three soil cores before planting in the three plantation sites

| Site | Soil type | pH | Total C  (g Kg^-1^) | Total N  (g Kg^-1^) | NH_4_  (mg Kg^-1^) | NO_3_  (mg Kg^-1^) | P  (mg Kg^-1^) | K  (mg Kg^-1^) | Ca  (mg Kg^-1^) | Mg  (mg Kg^-1^) |
| --- | --- | --- | --- | --- | --- | --- | --- | --- | --- | --- |
| Watford | Loam | 4.7 | 39 | 2.9 | 4 | 2.5 | 9.3 | 36 | 1022 | 28 |
| Asselin | Loam | 3.8 | 42 | 2.4 | 15 | 5 | 4 | 50 | 67 | 16 |
| Deville | Clay loam | 3.4 | 69 | 2.6 | 7 | 1 | 9 | 97 | 296 | 49 |

Supplementary Table 2: Seedlings characteristics before planting (means ± SD)

| SO | Height | | N (mg g^-1^) | P (mg g^-1^) | K (mg g^-1^) | Ca (mg g^-1^) | Mg (mg g^-1^) |
| --- | --- | --- | --- | --- | --- | --- | --- |
|  |  | |  |  |  |  |  |
|  | | **Needle** | | | | | |
| SO1 | 39.3(3.1) | | 15.0 (0.2) | 1.8 (0.1) | 5.0 (0.1) | 3.2 (0.3) | 1.0 (0.1) |
| SO2 | 40.8(3.1) | | 16.1 (0.8) | 2.0 (0.1) | 4.7 (0.3) | 3.4 (0.7) | 1.1 (0.1) |
| SO3 | 38.6(2.8) | | 16.3 (0.3) | 2.1 (0.1) | 5.3 (0.2) | 3.9 (0.4) | 1.0 (0.1) |
| SO4 | 35.8(2.7) | | 15.8 (0.8) | 2.1 (0.2) | 5.2 (0.4) | 3.3 (0.2) | 1.0 (0.1) |
| SO5 | 36.1(3.1) | | 16.8 (0.2) | 2.3 (0.1) | 4.7 (0.2) | 3.7 (0.2) | 1.1 (0.1) |
| SO6 | 40.1(3.1) | | 16.0 (0.8) | 2.2 (0.1) | 5.0 (0.3) | 3.4 (0.2) | 1.0 (0.1) |
|  | | **Root** | | | | | |
| SO1 |  | | 15.1 (0.4) | 2.8 (0.2) | 4.7 (0.3) | 3.4 (0.1) | 1.3 (0.2) |
| SO2 |  | | 15.9 (0.9) | 2.7 (0.3) | 3.8 (0.4) | 3.8 (0.1) | 1.2 (0.2) |
| SO3 |  | | 18.5 (1.0) | 2.9 (0.3) | 3.4 (0.5) | 3.9 (0.3) | 1.3 (0.2) |
| SO4 |  | | 17.2 (1.5) | 2.9 (0.3) | 3.4 (0.6) | 3.7 (0.4) | 1.2 (0.2) |
| SO5 |  | | 17.1 (0.5) | 2.4 (0.2) | 2.6 (0.4) | 3.6 (0.2) | 1.0 (0.1) |
| SO6 |  | | 10.7 (0.8) | 2.5 (0.2) | 3.2 (0.3) | 3.6 (0.2) | 1.1 (0.1) |
|  | | **Shoot** | | | | | |
| SO1 |  | | 1.0 (0.4) | 1.7 (0.1) | 5.4 (0.2) | 2.1 (0.1) | 1.0 (0.1) |
| SO2 |  | | 1.1 (0.1) | 1.8 (0.1) | 5.4 (0.6) | 2.4 (0.1) | 1.0 (0.1) |
| SO3 |  | | 1.2 (1.1) | 2.1 (0.2) | 5.8 (0.5) | 2.7 (0.3) | 1.1 (0.1) |
| SO4 |  | | 1.2 (0.4) | 2.0 (0.1) | 5.8 (0.3) | 2.5 (0.1) | 1.0 (0.1) |
| SO5 |  | | 1.1 (0.5) | 2.0 (0.1) | 5.4 (0.1) | 2.6 (0.2) | 1.1 (0.1) |
| SO6 |  | | 1.0 (0.9) | 1.9 (0.1) | 5.5 (0.4) | 2.5 (0.2) | 1.0 (0.1) |

Data are presented as mean (standard deviation). N=15.
